# Diet quality in the population of Norway and Poland: differences in the availability and consumption of food considering national nutrition guidelines and food market

**DOI:** 10.1186/s12889-021-10361-3

**Published:** 2021-02-09

**Authors:** Ewelina Janowska-Miasik, Anna Waśkiewicz, Anna Maria Witkowska, Wojciech Drygas, Maria Wik Markhus, Małgorzata Elżbieta Zujko, Marian Kjellevold

**Affiliations:** 1 Institute of Marine Research, P.O. Box 1870, NO-5817 Bergen, Nordnes Norway; 2 Department of Epidemiology, Cardiovascular Disease Prevention and Health Promotion, National Institute of Cardiology, Alpejska 42, 04-628 Warsaw, Poland; 3 Department of Food Biotechnology, Faculty of Health Sciences, Medical University of Bialystok, Szpitalna 37, 15-295 Bialystok, Poland; 4 Department of Social and Preventive Medicine, Faculty of Health Sciences, Medical University of Lodz, Hallera 1, 90-001 Lodz, Poland

**Keywords:** Diet, Dietary patterns, Population study, Norway, Poland, Food balance, Nutrients, Recommended intake (RI), Food-based dietary guidelines (FBDG), 24-h recall

## Abstract

**Background:**

Adequate nutrition is a public health challenge due to the increase in the incidence of diet-related diseases. The aim of this study was to examine food and nutrient intakes in the light of the current dietary guidelines of Poland and Norway. This is a suitable model for studying the diet quality in countries with different degrees of government intervention in the food market, which may affect food diversity available for citizens.

**Methods:**

The food diversity on the market was assessed using national food balance sheets. To show the actual food and nutrient intake within countries, data from 24-h recalls from the national surveys, NORKOST 3 from Norwegians and WOBASZ II from Poles, were used. In order to evaluate whether dietary patterns comply with nutritional and dietary recommendations, the Norwegian and Polish recommendations for nutrition and the national food based dietary guidelines (FBDGs) were analyzed.

**Results:**

Significant differences between the national supplies for most food products were found. Only subtle differences in the national FBDGs and nutritional recommendations were found. Low compliance with the national FBDGs for milk, fish and sugar consumption in Poland was observed. The intakes of most nutrients were in line with the countries’ nutrition recommendations. The intakes of folate and vitamin D by both genders and the intake of iron among women, were inadequate in both countries. Calcium and magnesium intakes were below the recommended intake among the adult population of Poland, additionally, insufficient intake of potassium and thiamine was found among Polish women.

**Conclusions:**

Despite the limited availability of certain food products on the market, the diet of Norwegians was better balanced in terms of food consumed and micronutrient intakes. The good supply of various groups of food has not, however, reduced the problem of widespread deficiency of vitamin D and folic acid in the diet, and action should be taken at national level to eliminate their inadequacy. In view of increasing risk of non-communicable diseases, low compliance with the dietary guidelines requires educational campaigns aimed at increasing dietary literacy in vulnerable groups.

**Supplementary Information:**

The online version contains supplementary material available at 10.1186/s12889-021-10361-3.

## Background

Nutrition is a cornerstone of sustainable development and one of the biggest global development challenges. Currently 88% of the countries in the world for which data are available are struggling with the problem of poor nutrition [1]. In addition, on a global scale, the world is not on track to achieve the United Nations Sustainable Development Goals [1]. A recent report concludes that substantial change is needed for the Nordic countries to meet the 2030 Agenda, and the increase in prevalence of overweight and obesity is mentioned as an example of poor diet [2].

One possible way to change this track is to translate these goals into a healthy diet through food-based nutrient-sensitive dietary recommendations, since diet is one of the most important elements influencing nutritional status. Furthermore, nutrition is usually considered to be a problem of knowledge and behavior, since the final choice of a diet is affected by personal factors [3]. Therefore, the level of nutrition literacy and role of education through guidelines can create demands for nutrient- rich food.

Nutrition recommendations focus on nutrient intakes, in particular by suggesting a reduction of total fat intake, and reduction in a saturated fat intake, and the elimination of trans- fats. They also take into account carbohydrate intake and limited intake of added sugars [4]. In order to transfer the knowledge from science-based recommendations to practical guidelines, food-based dietary guidelines (FBDG) have been developed. According to the European Food Safety Authority (EFSA), FBDGs are science-based easy-to-understand healthy nutrition recommendations for consumers. FBDGs are specific to regions and countries, where they are culturally acceptable and feasible to implement [5]. These guidelines provide advice on foods, food groups and dietary patterns that aim to provide residents with the necessary nutrients to promote general health and prevent chronic diseases [4, 5]. Taking into account the above, experts from Norway and Poland have developed appropriate recommendations [6–8].

However, to ensure that the population’s dietary patterns are consistent with such guidelines, consumers should have access to appropriate food products [9]. Ensuring the availability to nutritious food is crucial and depends on how markets function at local level. ‘What to eat’ it is an individual decision, but it is the responsibility of the government to ensure a nutritionally adequate diet [10].

The diversity and quality of food available on the market, its prices and the information that consumers receive about different products depend on national policies and regulations [11] and are dependent on social and cultural norms and values [12]. Differences in governmental intervention exist even in countries that are part of the European Union (EU) single market.

The European Economic Area (EEA) aims in applying the freedom of goods, services, persons and capital within the EU internal market. Norway, a non-EU member, has an individual agreement on trade of processed agricultural products (Protocol 3) [13]. In addition, Norway and the EU have concluded a bilateral agreement on trade of primary agricultural products (Article 19) [14]. However, the Norwegian market is not as open as the Polish market for agricultural products and processed food. As a result, the variety of products available to Norwegian consumers is somewhat limited.

The aim of this study was to examine food and nutrient intakes in the light of the current dietary guidelines of Poland and Norway. This is a suitable model for studying the diet quality in countries with different degrees of government intervention in the food market, which may affect food diversity available for citizens.

## Methods

### Analysis of national food balance sheets

Data on selected foodstuffs in total and per capita measures were collected from food balance sheets in order to compare food availability between the two countries, and to determine trends in the overall national food supply. These data are based on production and import statistics, with deductions for export, animal feed, seeds and other non-food uses and relate to the availability of food at wholesale level. The source of information was the data presented by the Central Statistical Office, Statistical Yearbook of Agriculture for the Polish population [15] and by the Norwegian Directorate for Health for the Norwegian population [16]. The analysis of food availability in Poland and Norway covered the years 2006–2016. The following nine food groups were analyzed: 1) milk and milk products, 2) poultry and meat, 3) fish and shellfish, 4) cereals including rice, 5) vegetables, 6) potatoes, 7) fruit and berries, 8) sugar and sweet products, 9) margarine, butter, oil, etc. (Table 1). Availability of milk and milk products is expressed as milk equivalent, i.e. it is converted into milk, both milk for direct consumption and milk for processed products, with appropriate conversion factors [17]. Availability of cereals was converted to flour using appropriate milling coefficients [18]. The figures for each product group are given in g/capita/day (Table 2).

**Table 1:** Food groups and related food products

Food groups	Products included in the group^**a**^
Milk and milk products	milk (whole, semi-skimmed, skimmed, condensed milk) milk-based beverages, sweet milk, curds, yoghurt, kefir, etc. soft cheese, hard cheese and other types of cheese, cream, sour cream and its substitutes, ice creams, milk-cream desserts
Poultry and meat	red meat: pork, beef, veal, other meat (mutton, horse meat, pork and beef offal), pork and beef smoked meats (hams, sirloins, sausages, etc) poultry meat: chicken, turkey, other poultry (duck, geese and poultry offal), poultry smoked meats (hams, sirloins, sausages, etc) and rabbit
Fish and shellfish	fresh fish, smoked and processed fish (canned fish, salted fish, other fish products)
Cereals, including rice	bread: light bread, wholemeal bread, rolls (various kinds of rolls, toasted bread), flour, pasta, cereal and rice, cereals (various types, also with toppings, bran, muesli)
Vegetables	fresh, frozen vegetables, processed vegetables, vegetable products and legumes
Potatoes	all potato dishes
Fruit and berries	fresh, frozen and processed fruits (e.g. jam, but excluding juices)
Sugar and sweet products	sugar, sweets: various types of cakes and biscuits, candies, chocolates, sweet bars, honey
Margarine, butter, oil etc.	animal fats added (butter, lard), vegetable fats added (soft and hard margarines, oils), and mixed fats added (blends of butter with margarine or oil)

^a^All products were treated equally during aggregation

**Table 2:** Food availability structure and trends - means from years 2006–2016

Food items	Mean													Trend		
	g/capita/day															
		2006	2007	2008	2009	2010	2011	2012	2013	2014	2015	2016	2006–2016	*p*- value		*p*-value
Milk and milk products	Norway^a^	479.2	482.7	477.0	453.7	451.5	443.0	437.3	422.5	412.0	402.7	403.0	441.0	≤0.001	↓	0.0000
	Poland^b^	482.2	490.4	498.6	517.8	517.8	531.5	528.8	564.4	561.6	583.6	608.2	534.2		↑	0.0000
Poultry and meat	Norway^a^	195.6	207.1	207.1	203.8	201.9	205.5	206.3	210.1	206.8	209.0	211.0	206.0	0.2	↑	0.0324
	Poland^b^	203.6	212.6	206.3	205.5	201.9	201.1	194.5	184.9	201.6	205.5	212.6	202.7		↓	0.6041
Fish and shellfish	Norway^a^	136.4	144.7	141.9	138.9	137.3	136.7	139.7	139.5	135.6	132.3	126.6	137.3	≤0.001	↓	0.0137
	Poland^b^	32.1	34.2	36.4	36.2	37.9	33.5	32.2	33.3	36.8	34.2	35.9	34.8		↓	0.9473
Cereals inc. rice	Norway^a^	243.3	246.3	242.5	236.7	235.3	228.8	224.7	224.9	223.3	225.5	229.0	223.9	≤0.001	↓	0.0003
	Poland^b^	320.5	312.3	306.8	304.1	295.9	295.9	295.9	295.9	290.4	282.2	282.2	298.6		↓	0.0000
Vegetables	Norway^a^	179.2	187.1	201.9	187.4	197.3	209.8	202.7	208.7	215.6	212.1	221.9	202.2	≤0.001	↑	0.0001
	Poland^b^	298.6	315.1	315.1	317.8	290.4	284.9	282.2	279.5	284.9	287.7	290.4	295.9		↓	0.0193
Potatoes	Norway^a^	196.4	192.3	209.8	160.5	169.6	159.5	171.2	162.5	179.7	140.5	169.3	173.7	≤0.001	↓	0.0281
	Poland^b^	331.5	331.5	323.3	317.8	301.4	304.1	304.1	279.4	276.7	273.9	265.7	301.4		↓	0.0000

^a^The Norwegian Directorate for Health; ^b^Polish Central Statistical Office

↓−Decreasing trend ↑−Increasing trend

A simple regression analysis was performed to determine the trend

Mann-Whitney’s U-test was used to test the differences, statistical significance was assessed for *p* < 0.05

### Determination of food consumption and nutrient intakes in individual diets from national dietary surveys

In order to show the actual nutrient and dietary intake by individuals in the countries, data from national surveys, NORKOST 3 [19, 20] for Norwegians and WOBASZ II [21, 22] for Poles, representative for each population by age and gender, were used.

In the NORKOST 3 survey, two randomly distributed 24-h recalls were used to assess the diet in a nationally representative sample (*N* = 5000) of men and women between 18 and 70 years of age randomly selected from the National Register [19]. The survey was conducted during the years 2011–2012 by the University of Oslo in collaboration with the Directorate of Health and the Food Safety Authority in Norway. Of the 5000 invited, 153 were unavailable for contact. In total, 1760 participants (849) men and (911) women) were included in the final study, resulting in a participation rate of 37% [19, 20].

WOBASZ II was a nationwide, cross-sectional study conducted on a randomly selected sample of 15.200 residents of 16 voivodships of Poland aged > 18 years. The sample was recruited from the National Register using a multistage sampling design. One thousand five hundred fifty-seven persons were not eligible. Out of eligible persons, 6170 were examined and the final response rate was 45.5%. Additional statistical analyses confirmed similar age distribution in the general Polish population and the study group, thus indicating that the WOBASZ II study was indeed representative for the general Polish population. To establish food consumption, WOBASZ II survey used 24-h recall methodology. The final analysis of dietary habits included 4979 respondents aged 18–69 after some subjects were excluded due to missing or unreliable diet recalls [21, 22].

In both countries, photographs and images of different sizes of food portions were used to facilitate quantitative estimation of food consumption. Table 1 shows the grouping of food products into nine categories that covered most of foods consumed. In this study, the individual food consumption is expressed as g/day. It takes into account the consumption of processed products without converting them into raw food, i.e. the consumption of milk, fermented milk drinks, cheeses and other milk products, is summed up for milk and milk products. The mean and standard deviation (SD) were calculated for the intake of each food group.

### Assessment of nutrient intakes and dietary patterns according to national nutrition and dietary recommendations

Norwegian and Polish nutrition recommendations (Additional file 1: Table S1, S2 and S3) and FBDGs (Additional file 1: Table S4) have been used to assess whether the dietary patterns are consistent with national nutrition and dietary recommendations. On the basis of consumption data in national surveys, the energy and nutrient content in the diet was calculated using the Polish and Norwegian food composition tables [23, 24]. The following conversion factors were used to calculate the energy intake: for fat 37 kJ/g, for protein and carbohydrates 17 kJ/g, for dietary fiber 8 kJ/g and for alcohol 29 kJ/g. The following official sources of dietary recommendations for Norway and Poland were used: Nordic Nutrition Recommendation [6], Recommendation about diet, nutrition and physical activity [7] for Norwegian population, and Nutrition standards for Polish population [8], which contains both nutrition recommendation and dietary guidelines. The data are presented as % energy intake (E%) for macronutrients and as recommended intake (RI) for certain micronutrients for adults (18–64 years) of both genders. The Recommended Dietary Allowance (RDA) was used as a cut-off value to assess the adequacy of nutrient intake, except for dietary fiber, vitamin D and E, potassium and sodium for which Adequate Intake (AI) was used. Added sugars were calculated for processed food with the exception of naturally occurring sugars (e.g. in vegetables, fruit, dairy products).

### Statistical analysis

The Student’s t-test and Mann-Whitney’s U-test were used to investigate the differences between consumption structures in Norway and Poland. Statistical significance was found at *p* < 0.05. A simple regression analysis was conducted to determine the trend.

The statistical analysis was conducted using Statistica 64, version 13.1 (Dell Software, Inc., Round Rock, TX, USA) and Statistical Analysis System (SAS), version 9.4 (SAS Institute Inc., Cary, NC, USA).

## Results

The comparison of data on the structure of food availability in Norway and Poland at the national level is presented in Table 2. More fish and fruits were available in Norway, while milk, cereals, vegetables, potatoes, sugar plus sweet products and margarine plus butter and other fats were more available on the Polish market. The overall food supply in Poland comply with the recommendations for vegetables and fruit as a total. In both countries the availability of fruit on the food market is increasing but not significantly. In addition, the availability of vegetables in Norway followed the same trend. In turn, the Polish vegetable supply shows a downward trend. The supply of milk and milk products in Poland was also adequate and supported by a significant increase in market availability. The supply of milk and milk products in Norway followed the opposite trend. The analysis also showed that in the analyzed period a significant decrease in the supply of sugar, margarine, butter and oil was observed in Norway as opposed to Poland, where the availability of sweets on the market increased significantly, while the increase in margarine, butter and oil was not significant. The availability of meat and poultry on the Norwegian market increased significantly between 2006 and 2016. In both countries a significant tendency towards a reduced supply of staple potatoes and cereals was observed. The Norwegian market was also characterized by a significant decrease in the availability of fish and shellfish, but still exceeding the recommended level.

The diet of adults in both countries is compatible with the food available on the market. Significant differences in food consumption between the countries were found for milk and milk products, fish and shellfish, vegetables, potatoes, margarine, butter, oil in all sex and age groups, as shown in Tables 3 and 4. Moreover, significant differences were found for all age groups of men with regard to the consumption of poultry and meat, and in women with regard to sugar and sweet products. In contrast to the food availability on the market, more milk and milk products were consumed by adults in Norway. In turn, all age-groups of Polish men and women aged 36–69 consumed significantly more meat and poultry products. In terms of fish consumption, it was significantly lower in Poland in all sex and age groups. In Norway, only women aged 18–35 had lower fish consumption than recommended, but it was still more than 2 times higher than in Polish women in the same age group. Furthermore, the mean intake of vegetables and fruit was adequate among the adult Polish population, but the proportions between them did not comply with the recommended. Sugar intake is one of the main targets of global reduction, but neither the Polish nor Norwegian dietary guidelines give any specific quantities recommended for consumption. It was therefore difficult to compare these food products data with the recommendations. The situation was similar for cereals, potatoes and margarine, butter and oil.

**Table 3:** Food intake among men (edible amount g/day) in Norway^a^ (*n* = 849) and Poland^b^ (*n* = 2267) by age groups

Food items	18–35		36–55		56–69	
	Norway *n* = 212	Poland *n* = 633	Norway *n* = 364	Poland *n* = 945	Norway *n* = 273	Poland *n* = 689
Milk and milk products (mean ± SD) median (25–75 percentile)	441 ± 390	198 ± 254^1^	430 ± 381	158 ± 203^1^	354 ± 282	170 ± 224^1^
	348 (160–605)	106 (30–282)	326 (139–642)	80 (24–239)	302 (108–550)	78 (20–250)
Poultry and meat (mean ± SD) median (25–75 percentile)	208 ± 152	242 ± 190^2^	180 ± 112	241 ± 186^1^	161 ± 119	212 ± 185^1^
	175 (106–271)	205 (105–330)	159 (99–251)	203 (116–322)	144 (82–217)	180 (90–294)
Fish and shellfish (mean ± SD) median (25–75 percentile)	53 ± 86	18 ± 58^1^	73 ± 88	23 ± 74^1^	107 ± 122	25 ± 74^1^
	0 (0–85)	0 (0–0)	36 (0–124)	0 (0–0)	75 (5–156)	0 (0–0)
Cereals inc. rice (mean ± SD) median (25–75 percentile)	320 ± 154	282 ± 156^2^	277 ± 125	259 ± 135^2^	229 ± 106	227 ± 110
	308 (206–414)	252 (174–352)	258 (189–336)	235 (161–330)	215 (152–284)	212 (150–290)
Vegetables (mean ± SD) median (25–75 percentile)	173 ± 116	254 ± 186^1^	148 ± 101	268 ± 189^1^	149 ± 104	281 ± 195^1^
	158 (94–221)	230 (120–350)	131 (71–285)	232 (133–374)	128 (81–193)	253 (146–376)
Potatoes (mean ± SD) median (25–75 percentile)	57 ± 70	292 ± 316^1^	78 ± 76	281 ± 271^1^	110 ± 84	278 ± 261^1^
	22 (0–98)	231 (0–461)	121 (70–184)	231 (0–461)	98 (55–163)	231 (0–461)
Fruit and berries (mean ± SD) median (25–75 percentile)	128 ± 134	164 ± 217^2^	163 ± 146	199 ± 271^2^	207 ± 169	218 ± 240
	101 (11–185)	100 (0–250)	129 (45–248)	125 (0–300)	175 (76–288)	150 (10–310)
Sugar and sweet prod. (mean ± SD) median (25–75 percentile)	55 ± 87	70 ± 81^2^	53 ± 63	73 ± 85^1^	53 ± 65	62 ± 78
	26 (3–78)	46 (10–100)	30 (5–78)	43 (10–105)	31 (5–81)	34 (8–87)
Margarine, butter, oil etc. (mean ± SD) median (25–75 percentile)	39 ± 26	56 ± 44^1^	40 ± 25	51 ± 36^1^	37 ± 29	45 ± 32^1^
	33 (20–50)	46 (27–70)	36 (21–51)	43 (27–66)	31 (17–51)	38 (23–59)

Highlighted numbers show statistical difference between countries within each age group:^1^ *p* ≤ 0.001; ^2^ *p* ≤ 0.05

^a^From NORKOST 3; ^b^From WOBASZ II

**Table 4:** Food intake among women (edible amount g/day) in Norway^a^ (*n* = 911) and Poland^b^ (*n* = 2712) by age groups

Food items	18–35		36–55		56–69	
	Norway n = 226	Poland *n* = 692	Norway *n* = 465	Poland *n* = 1168	Norway *n* = 220	Poland *n* = 852
Milk and milk products (mean ± SD) median (25–75 percentile)	312 ± 264	198 ± 192^1^	256 ± 226	179 ± 167^1^	258 ± 236	182 ± 187^1^
	244 (125–445)	150 (50–288)	200 (83–369)	140 (46–260)	186 (97–395)	125 (42–262)
Poultry and meat (mean ± SD) median (25–75 percentile)	122 ± 83	132 ± 111	119 ± 77	132 ± 113^2^	105 ± 74	138 ± 117^1^
Fish and shellfish (mean ± SD) median (25–75 percentile)	113 (60–162)	117 (44–185)	107 (63–164)	121 (40–189)	92 (50–148)	122 (35–202)
	28 ± 61	13 ± 48^1^	58 ± 74	15 ± 54^1^	68 ± 72	16 ± 57^1^
	0 (0–59)	0 (0–0)	30 (0–91)	0 (0–0)	43 (0–118)	0 (0–0)
Cereals inc. rice (mean ± SD) median (25–75 percentile)	199 ± 101	176 ± 104^2^	182 ± 86	171 ± 101^2^	154 ± 77	160 ± 75
	177 (131–253)	156 (107–223)	168 (120–231)	154 (105–210)	142 (103–191)	152 (108–203)
Vegetables (mean ± SD) median (25–75 percentile)	135 ± 89	212 ± 150^1^	163 ± 108	245 ± 168^1^	161 ± 111	253 ± 163^1^
	125 (71–177)	195 (102–291)	144 (96–210)	215 (125–334)	140 (92–199)	228 (130–359)
Potatoes (mean ± SD) median (25–75 percentile)	42 ± 60	177 ± 204^1^	50 ± 58	204 ± 212^1^	58 ± 52	203 ± 201^1^
	7 (0–72)	117 (0–292)	35 (0–82)	179 (0–328)	50 (0–93)	200 (0–308)
Fruit and berries (mean ± SD) median (25–75 percentile)	160 ± 136	209 ± 228^1^	185 ± 139	223 ± 236^1^	226 ± 155	246 ± 240
	138 (56–239)	150 (15–300)	166 (75–264)	175 (41–305)	205 (105–301)	197 (81–340)
Sugar and sweet prod. (mean ± SD) median (25–75 percentile)	56 ± 60	68 ± 76^2^	51 ± 51	68 ± 77^1^	51 ± 55	61 ± 72^2^
	40 (13–89)	45 (12–100)	37 (10–75)	45 (10–100)	37 (8–70)	38 (9–85)
Margarine, butter, oil etc. (mean ± SD) median (25–75 percentile)	23 ± 19	35 ± 26^1^	24 ± 19	36 ± 25^1^	26 ± 20	34 ± 22^1^
	19 (10–32)	30 (18–44)	20 (10–33)	31 (19–47)	22 (13–34)	30 (18–45)

Highlighted numbers show statistical difference between countries within each age group:^1^ *p* ≤ 0.001; ^2^ *p* ≤ 0.05

^a^From NORKOST 3; ^b^From WOBASZ II

The intake of energy among men and women is shown in Tables 5 and 6, respectively. Polish adults were characterized by a lower percentage of energy coming from proteins compared to Norwegian adults, and the differences between the average values were significant. Moreover, in both populations studied, the level of compliance with the countries’ recommendations concerning energy from protein, was very high. The opposite was true for fats, which provided a higher energy supply for all age-groups of Polish men, and for Polish women aged 18–55. In addition, the total energy intake from fats was higher in Polish men and in women aged 18–55 than that recommended for adult Poles. The intake of energy supplied by saturated fatty acids was much above the level recommended by both countries, and the difference between the average in Poland and Norway was not significant only in the group of women aged 36–55 and in the oldest group of men analyzed. The intake of energy from monounsaturated fatty acids (MUFA) and polyunsaturated fatty acids (PUFA) is consistent with the recommendations, but only the differences between the average MUFA values for adults of both genders were significant. The energy from PUFA varies significantly in the 36–55 age group for men and in the women aged 18–35 and 56–69. Norwegian men, except the 18–35 age group, were characterized by lower intake of energy from carbohydrates than it is recommended. The same was observed in all age groups of Polish men. Lower energy intake from carbohydrates than it is recommended was also observed among Norwegian women aged 36–69. The differences between the average values were significant among women aged 36–69 and men aged 18–35 and 56–69.

**Table 5:** Comparison of energy structure from nutrients among men in Norway^a^ (*n* = 849) and Poland^b^ (*n* = 2267) by age groups% energy from nutrients

% energy from nutrients	18–35		36–55		56–69	
	Norway *n* = 212	Poland *n* = 633	Norway *n* = 364	Poland *n* = 945	Norway *n* = 273	Poland *n* = 689
Protein, E% (mean ± SD) median (25–75 percentile)	17.4 ± 3.9	15.3 ± 3.9^1^	17.7 ± 3.7	15.3 ± 3.7^1^	18.1 ± 3.4	15.6 ± 4^1^
	17.1 (15.0–19.1)	15 (12.6–17.4)	17.2 (15.0–20.0)	14.8 (12.9–17.3)	17.7 (15.5–20.3)	15.1 (13–17.9)
Fat, E% (mean ± SD) median (25–75 percentile)	33.0 ± 7.0	38.4 ± 8.3^1^	34.8 ± 6.7	37.9 ± 8.3^1^	34.6 ± 8.0	37 ± 8.5^1^
	33.0 (28.4–37.3)	38.7 (33.3–43.7)	34.8 (30.7–39.2)	37.2 (32.4–43.5)	33.8 (29.5–39.6)	36.8 (31.7–42.1)
SFA, E% (mean ± SD) median (25–75 percentile)	12.8 ± 3.1)	14.5 ± 4.3^1^	13.2 ± 3.1	13.8 ± 4.3^2^	13.3 ± 3.7	13.5 ± 4.4
	12.7 (10.6–14.6)	14.1 (11.6–17.3)	13.2 (10.9–15.5)	13.4 (10.7–16.6)	13.2 (10.7–16.0)	12.9 (10.6–16)
MUFA, E% (mean ± SD) median (25–75 percentile)	11.2 ± 3.0	15.4 ± 4.2^1^	11.8 ± 2.8	15.3 ± 4.3^1^	11.6 ± 3.4	14.9 ± 4.4^1^
	10.7 (9.1–12.7)	15.1 (12.4–17.9)	11.6 (9.8–13.5)	15.1 (12.4–18)	11.2 (9.4–13.7)	14.5 (12–17.6)
PUFA, E% (mean ± SD) median (25–75 percentile)	5.9 ± 2.1	5.8 ± 2.5	6.5 ± 2.2	6.1 ± 2.5^2^	6.2 ± 2.3	6 ± 2.6
	5.5 (4.6–7.2)	5.4 (4–7.1)	6.2 (5.0–7.8)	5.7 (4.3–7.5)	5.9 (4.5–7.6)	5.7 (4.2–7.2)
Carbohydrates^c^, E% (mean ± SD) median (25–75 percentile)	45.2 ± 7.8	43.6 ± 9.9^2^	43.4 ± 7.7	43.6 ± 10.2	42.2 ± 8.0	44.1 ± 9.8^2^
	45.5 (41.3–50.2)	43.7 (37.7–50.0)	44.0 (38.3–48.4)	44.3 (37.9–50.0)	42.9 (36.9–47.6)	44.9 (38.5–50.0)
Free sugars, E% (mean ± SD) median (25–75 percentile)	8.8 ± 6.9	10.3 ± 9.9^2^	7.0 ± 5.6	11.6 ± 11.8^1^	6.2 ± 4.6	10.7 ± 11.2^1^
	7.0 (4.1–12.2)	8.1 (2–15.3)	5.7 (3.1–9.6)	8 (2.5–17.2)	5.3 (2.9–8.8)	7.1 (1.7–15.4)

*SFA* Saturated fatty acids, *MUFA* Monounsaturated fatty acids, *PUFA* Polyunsaturated fatty acids

Highlighted numbers shows statistical difference between countries within each age group:^1^ *p* ≤ 0.001; ^2^ *p* ≤ 0.05

^a^From NORKOST 3; ^b^From WOBASZ II; ^c^The % E of carbohydrates was calculated without fiber and alcohol

**Table 6:** Comparison of energy structure from nutrients among women in Norway^a^ (*n* = 911) and Poland^b^ (*n* = 2712) by age groups

% energy from nutrients	18–35		36–55		56–69	
	Norway *n* = 226	Poland *n* = 692	Norway *n* = 465	Poland *n* = 1168	Norway *n* = 220	Poland *n* = 852
Protein, E% (mean ± SD) median (25–75 percentile)	16.9 ± 3.9	15.0 ± 4.0^1^	17.8 ± 3.6	15 ± 4.1^1^	17.9 ± 3.4	15.4 ± 4.1^1^
	16.6 (14.1–19.0)	14.5 (12.5–17)	17.5 (15.3–19.9)	14.4 (12.3–16.9)	17.8 (15.4–19.9)	14.8 (12.6–17.6)
Fat, E% (mean ± SD) median (25–75 percentile)	33.1 ± 7.1	35.6 ± 8.5^1^	34.4 ± 7.1	35.6 ± 8.4^2^	35.7 ± 7.6	34.9 ± 8.1
	32.5 (28.2–38.5)	35.1 (30–41.1)	34.4 (29.8–38.6)	35.7 (30.1–40.8)	35.3 (30.2–40.1)	34.7 (29.5–40.2)
SFA, E% (mean ± SD) median (25–75 percentile)	12.8 ± 3.2	13.9 ± 4.5^1^	13.2 ± 3.4	13.3 ± 4.3	13.5 ± 3.5	12.9 ± 4.2^2^
	12.8 (10.4–15.0)	13.5 (10.8–16.7)	13.1 (10.8–15.4)	13 (10.2–15.9)	13.0 (10.9–15.9)	12.5 (9.9–15.6)
MUFA, E% (mean ± SD) median (25–75 percentile)	11.2 ± 2.9	13.7 ± 4.2^1^	11.6 ± 3.1	13.8 ± 4.2^1^	12.1 ± 3.5	13.5 ± 4.2^1^
	11.0 (9.1–13.0)	13.5 (10.7–16.4)	11.4 (9.4–13.5)	13.6 (10.9–16.3)	11.6 (9.8–13.3)	13.2 (10.8–16.1)
PUFA, E% (mean ± SD) median (25–75 percentile)	5.9 ± 2.4	5.4 ± 2.5^2^	6.1 ± 2.2	5.9 ± 3.1	6.5 ± 2.4	5.9 ± 2.8^1^
	5.5 (4.2–6.9)	4.9 (3.7–6.5)	5.9 (4.5–7.5)	5.3 (3.9–7.2)	6.1 (4.7–7.9)	5.3 (3.9–7.3)
Carbohydrates^c^, E% (mean ± SD) median (25–75 percentile)	46.7 ± 7.4	47.1 ± 93	43.3 ± 8.0	47.3 ± 9.7^1^	41.3 ± 8.0	47.3 ± 9.3^1^
	46.9 (42.1–52.1)	47.5 (41.4–53.2)	43.7 (38.2–48.6)	49.4 (40.1–53.1)	42.0 (36.8–46.1)	49.7 (41.5–53.5)
Free sugars, E%(mean ± SD) median (25–75 percentile)	9.3 ± 6.2	14.2 ± 13.4^1^	7.0 ± 4.8	14.4 ± 13.7^1^	6.3 ± 4.2	13.3 ± 13.3^1^
	7.9 (5.0–12.5)	11 (3.6–21.8)	6 (3.4–9.0)	11 (3–22.3)	5.7 (3.3–8.4)	9.9 (2.1–19.9)

*SFA* Saturated fatty acids, *MUFA* Monounsaturated fatty acids, *PUFA* Polyunsaturated fatty acids

Highlighted numbers shows statistical difference between countries within each age group:^1^ *p* ≤ 0.001; ^2^ *p* ≤ 0.05

^a^From NORKOST 3; ^b^From WOBASZ II; ^c^The % E of carbohydrates was calculated without fiber and alcohol

Although the average intake of most nutrients varied significantly between countries in each age group, most of them complied with the countries’ nutrient recommendations. However, the degree of compliance was variable, as presented in Tables 7 and 8. The intakes of folate and vitamin D in both adult populations were not sufficient. Adult women from both populations were also characterized by insufficient iron intake. In addition, the intake of dietary fiber was only balanced in Norwegian men. Poor compliance with the recommendations was found in each age group with regard to calcium and magnesium intake by both genders of Polish population. Moreover, the intake of potassium was not consistent with the recommendation among all age groups of Polish women and in Polish men aged 56–69. The intake of thiamine among Polish women has also not reached the Polish nutritional recommendations.

**Table 7:** Energy and nutrients intake (per person per day) among men in Norway^a^ (*n* = 849) and Poland^b^ (*n* = 2267) by age groups

Energy/nutrients	18–35		36–55		56–69	
	Norway *n* = 212	Poland *n* = 633	Norway *n* = 364	Poland *n* = 945	Norway *n* = 273	Poland *n* = 689
Energy, MJ (mean ± SD) median (25–75 percentile)	12.24 ± 3.92	10.85 ± 4.48^1^	10.80 ± 3.14	10.06 ± 3.80^1^	9.89 ± 2.94	8.96 ± 3.51^1^
	11.75 (9.77–14.51)	10.15 (7.72–13.57)	10.52 (8.54–12.83)	9.38 (7.41–12.28)	9.55 (7.88–11.68)	8.62 (6.53–10.83)
Cholesterol, mg (mean ± SD) median (25–75 percentile)	420 ± 266	405 ± 275	392 ± 209	358 ± 227^2^	385 ± 206	317 ± 212^1^
	363 (245–503)	326 (213–536)	348 (243–439)	303 (194–459)	351 (238–489)	267 (175–399)
Dietary fibre, g (mean ± SD) median (25–75 percentile)	28 ± 11	21 ± 10^1^	26 ± 11	21 ± 9^1^	26.1 ± 10.3	21 ± 9^1^
	26 (20–34)	20 (14–27)	25 (19–31)	20 (15–27)	24.3 (18.8–31.8)	20 (15–26)
Vitamin A, RAE (mean ± SD) median (25–75 percentile)	1030 ± 887	1255 ± 2206^2^	948 ± 567	1209 ± 2186^1^	1036 ± 1309	1119 ± 2178
	811 (561–1227)	884 (539–1404)	871 (573–1167)	864 (530–1307)	805 (572–1189)	830 (525–1220)
Vitamin D, μg (mean ± SD) median (25–75 percentile)	5.6 ± 5.2	4.1 ± 4.6^1^	6.6 ± 5.2	4.2 ± 4.4^1^	7.6 ± 6.6	4.2 ± 5.4^1^
	4.1 (2.6–7.9)	3.0 (1.7–4.9)	5.6 (3.4–8.2)	3.1 (1.9–4.9)	5.8 (3.4–9.9)	2.8 (1.6–4.7)
Vitamin E, mg (mean ± SD) median (25–75 percentile)	12.6 ± 5.7	13.3 ± 7.8	12.1 ± 4.8	12.6 ± 7.0	12.0 ± 5.8	11.8 ± 7.3
	11.7 (8.4–15.8)	11.6 (7.7–16.8)	11.3 (8.5–14.4)	11.4 (7.6–15.9)	11.0 (8.1–14.7)	10.5 (7.3–14.7)
Thiamine, mg (mean ± SD) median (25–75 percentile)	2.0 ± 0.8	1.6 ± 0.9^1^	1.9 ± 0.7	1.5 ± 0.8^1^	1.7 ± 0.6	1.4 ± 0.7^1^
	1.9 (1.5–2.4)	1.4 (1.0–2.0)	1.8 (1.4–2.3)	1.4 (1.0–1.9)	1.6 (1.3–2.1)	1.2 (0.9–1.8)
Riboflavin, mg (mean ± SD) median (25–75 percentile)	2.3 ± 0.9	1.8 ± 1.0^1^	2.2 ± 0.8	1.7 ± 0.9^1^	2.0 ± 0.7	1.6 ± 0.9^1^
	2.2 (1.7–2.8)	1.6 (1.2–2.2)	2.0 (1.6–2.6)	1.5 (1.1–2.0)	1.9 (1.4–2.3)	1.4 (1.0–1.9)
Vitamin B_6_, mg (mean ± SD) median (25–75 percentile)	2.1 ± 1.0	2.3 ± 1.0^2^	1.9 ± 0.7	2.1 ± 0.9^1^	1.8 ± 0.7	2.0 ± 0.8^1^
	2.0 (1.5–2.5)	2.2 (1.5–2.9)	1.8 (1.4–2.3)	2.1 (1.5–2.6)	1.7 (1.3–2.1)	1.9 (1.4–2.5)
Vitamin B_12_, μg (mean ± SD) median (25–75 percentile)	8.8 ± 6.0	4.4 ± 5.9^1^	8.8 ± 6.1	4.4 ± 6.2^1^	9.0 ± 11.2	4.2 ± 7.7^1^
	7.4 (4.9–11.0)	3.2 (2.1–4.7)	7.2 (5.3–10.7)	2.8 (1.9–4.5)	7.3 (4.9–10.5)	2.6 (1.6–4.1)
Folate, μg (mean ± SD) median (25–75 percentile)	307 ± 117	282 ± 118^2^	272 ± 93	266 ± 111	270 ± 102	254 ± 134^2^
	289 (225–376)	268 (197–348)	263 (202–325)	253 (191–324)	256 (196329)	237 (183–303)
Vitamin C, mg (mean ± SD) median (25–75 percentile)	103 ± 77	90 ± 80^2^	99 ± 93	83 ± 77^1^	114 ± 83	84 ± 69^1^
	82 (45–141)	68 (36–115)	81 (48–132)	64 (33–110)	96 (50–148)	68 (37–104)
Calcium, mg (mean ± SD) median (25–75 percentile)	1182 ± 587	720 ± 546^1^	1058 ± 526	576 ± 395^1^	899 ± 383-	515 ± 342^1^
	1063 (780–1471)	544 (353–902)	945 (697–1346)	478 (304–733)	862 (6061132)	432 (269–649)
Iron, mg (mean ± SD) median (25–75 percentile)	13.7 ± 5.3	13.0 ± 6.2	12.4 ± 4.2	12.9 ± 6.1	11.9 ± 3.9	11.9 ± 5.5
	13.2 (10.1–16.6)	11.9 (9.1–15.4)	11.9 (9.5–14.5)	11.9 (9.0–15.2)	11.8 (9.2–14.2)	11 (8.7–14.2)
Magnesium, mg (mean ± SD) median (25–75 percentile)	457 ± 158	320 ± 131^1^	446 ± 143	309 ± 116^1^	419 ± 129	284 ± 110^1^
	439 (346–547)	302 (224–387)	426 (344–527)	294 (232–370)	399 (239–492)	272 (213–338)
Potassium, g (mean ± SD) median (25–75 percentile)	4.24 ± 1.41	3.68 ± 1.57^1^	4.25 ± 1.25	3.55 ± 1.36^1^	4.26 ± 1.25	3.39 ± 1.27^1^
	4.16 (3.16–5.11)	3.62 (2.54–4.55)	4.11 (3.39–4.97)	3.44 (2.57–4.36)	4.11 (3.41–5.15)	3.29 (2.54–4.14)

Highlighted numbers shows statistical difference between countries within each age group:^1^ *p* ≤ 0.001;^2^ *p* ≤ 0.05

^a^From NORKOST 3; ^b^From WOBASZ II

**Table 8:** Energy and nutrients intake (per person per day) among women in Norway^a^ (*n* = 911) and Poland^b^ (*n* = 2712) by age groups

Energy/nutrients	18–35		36–55		56–69	
	Norway *n* = 226	Poland *n* = 692	Norway *n* = 465	Poland *n* = 1168	Norway *n* = 220	Poland *n* = 852
Energy, MJ (mean ± SD) median (25–75 percentile)	8.37 ± 2.48	7.38 ± 2.88^1^	8.04 ± 2.37	7.22 ± 2.79^1^	7.49 ± 2.25	6.86 ± 2.46^1^
	8.37 (66.2–97.4)	7.10 (5.35–9.14)	8.02 (6.43–9.47)	6.83 (5.35–8.59)	7.17 (8.89–5.92)	6.65 (5.13–8.22)
Cholesterol, mg (mean ± SD) median (25–75 percentile)	258 ± 169	242 ± 149	311 ± 172	242 ± 163^1^	317 ± 174	233 ± 147^1^
	217 (158–316)	204 (133–317)	276 (183–403)	206 (131–300)	273 (181–422)	204 (133–296)
Dietary fibre, g (mean ± SD) median (25–75 percentile)	21.9 ± 8.4	17 ± 7^1^	22.4 ± 8.2	18 ± 8^1^	22.3 ± 8.2	18 ± 8^1^
	21 (16–26)	16 (12–21)	21.2 (16.8–26.7)	17 (13–22)	20.8 (17.1–26.3)	17 (13–22)
Vitamin A, RAE (mean ± SD) median (25–75 percentile)	709 ± 441	956 ± 1381^1^	794 ± 546	907 ± 1138^2^	767 ± 446	1066 ± 1979^1^
	605 (444–904)	703 (428–1057)	677 (481–915)	689 (445–1035)	666 (513–907)	722 (438–1056)
Vitamin D, μg (mean ± SD) median (25–75 percentile)	4.1 ± 3.4	2.4 ± 2.6^1^	4.9 ± 4.5	2.7 ± 3.5^1^	5.4 ± 4.6	2.6 ± 2.9^1^
	3.2 (1.8–5.5)	1.7 (1.0–2.8)	3.9 (2.1–6.1)	1.9 (1.1–3.1)	4.3 (2.4–7.1)	1.9 (1.2–3.0)
Vitamin E, mg (mean ± SD) median (25–75 percentile)	9.4 ± 4.3	9.3 ± 5.4	10.2 ± 4.3	10.0 ± 5.8	10.3 ± 4.3	9.7 ± 5.4
	8.4 (6.3–11.6)	8.2 (5.6–11.8)	9.8 (7.4–12.3)	8.8 (6.1–12.3)	9.8 (7.4–12.2)	8.6 (5.8–12.2)
Thiamine, mg (mean ± SD) median (25–75 percentile)	1.4 ± 0.5	1.0 ± 0.5^1^	1.4 ± 0.5	1.0 ± 0.5^1^	1.3 ± 0.4	1.0 ± 0.5^1^
	1.3 (1.1–1.7)	0.9 (0.7–1.2)	1.3 (1.1–1.7)	0.9 (0.7–1.3)	1.3 (1.0–1.6)	0.9 (0.7–1.2)
Riboflavin, mg (mean ± SD) median (25–75 percentile)	1.6 ± 0.6	1.3 ± 0.6^1^	1.6 ± 0.6	1.3 ± 0.6^1^	1.5 ± 0.6	1.3 ± 0.7^1^
	1.6 (1.2–2.1)	1.2 (0.9–1.6)	1.6 (1.2–1.9)	1.2 (0.9–1.6)	1.4 (1.1–1.7)	1.2 (0.9–1.6)
Vitamin B_6_, mg (mean ± SD) median (25–75 percentile)	1.5 ± 0.5	1.6 ± 0.7	1.5 ± 0.5	1.6 ± 0.7^2^	1.4 ± 0.5	1.6 ± 0.7^1^
	1.4 (1.1–1.8)	1.5 (1.1–2.0)	1.5 (1.2–1.8)	1.5 (1.1–2,0)	1.4 (1.1–1.7)	1.5 (1.1–1.9)
Vitamin B_12_, μg (mean ± SD) median (25–75 percentile)	5.6 ± 3.2	2.8 ± 3.2^1^	6.1 ± 3.8	2.8 ± 3.3^1^	6.2 ± 4.0	3.1 ± 5.0^1^
	4.9 (3.4–7.3)	2.2 (1.4–3.2)	5.1 (3.5–8.0)	2 (1.4–3.1)	5.9 (3.4–8.1)	2.1 (1.3–3.2)
Folate, μg (mean ± SD) median (25–75 percentile)	230 ± 85	216 ± 89^2^	234 ± 83	221 ± 91^1^	227 ± 94	219 ± 91
	220 (171–289)	205 (156–264)	220 (178–276)	207 (160–274)	211 (167–270)	207 (158–263)
Vitamin C, mg (mean ± SD) median (25–75 percentile)	102 ± 72	85 ± 742^2^	113 ± 71	88 ± 79^1^	113 ± 70	85 ± 67
	87 (45–139)	64 (36–111)	99 (58–147)	67 (39–112)	100 (65–153)	66 (42–110)
Calcium, mg (mean ± SD) median (25–75 percentile)	855 ± 379	573 ± 373^1^	806 ± 355	520 ± 318^1^	771 ± 356	478 ± 294^1^
	822 (565–1095)	480 (302–744)	762 (548–1009)	466 (302–654)	715 (506–994)	410 (258–615)
Iron, mg (mean ± SD) median (25–75 percentile)	9.9 ± 3.4	9.4 ± 4.1	10.1 ± 3.6	9.5 ± 3.9^2^	9.5 ± 3.3	9.6 ± 4.6
	9.5 (7.2–11.9)	8.7 (6.7–11.1)	9.6 (7.7–12.1)	8.9 (7.0–11.2)	9.2 (7.3–11.3)	8.8 (7.0–11.1)
Magnesium, mg (mean ± SD) median (25–75 percentile)	337 ± 116	246 ± 94^1^	356 ± 109	249 ± 91^1^	333 ± 105	237 ± 94^1^
	323 (256–397)	234 (182–294)	345 (278–424)	237 (186–300)	315 (261–389)	225 (178–282)
Potassium, g (mean ± SD) median (25–75 percentile)	3.32 ± 0.95	2.87 ± 1.13^1^	3.45 ± 1.00	2.95 ± 1.09^1^	3.38 ± 0.98	2.90 ± 1.10^1^
	3.23 (2.49–3.84)	2.71 (2.09–3.56)	3.37 (2.76–4.06)	2.88 (2.22–3.59)	3.18 (2.72–3.94)	2.84 (2.15–3.49)

Highlighted numbers shows statistical difference between countries within each age group:^1^ *p* ≤ 0.001;^2^ *p* ≤ 0.05

^a^From NORKOST 3; ^b^From WOBASZ II

## Discussion

Norway and Poland are two similar economies that differ in the scale of governmental intervention in the food market. This study examines the influence of government intervention on the structure of foods available for consumption and dietary intake by populations of Norway and Poland, and their compliance with dietary recommendations. Although both countries are a part of the EEA Agreement, Norway does not participate in the common agricultural policy or the common fisheries policy and protects its domestic production from global competition. Most of agricultural and fisheries goods originating from Norway do not benefit from completely free trade, and most agricultural and fisheries goods are excluded from the single market [25]. Moreover, high tariffs and quantitative restrictions limit entry of competing products. In addition, in order to maintain agricultural production, the Norwegian government subsidizes 60% of farmer’s income, while a farmer in Poland can expect state subsidies amounting to 20% of their annual income [26, 27]. High agricultural subsidies are justified in protected markets, but lead to less variety of available products, because the country is not able to produce more from the point of diversity [28]. The Norwegian food market is therefore characterized by a very narrow choice of food products available for consumers [29]. On the contrary, the Polish food market, which in a short period of time underwent major transformations (from a centrally controlled economy with food shortages on the market including food rationing to an open economy) is characterized by a large variety of available products. Additionally, in Poland, since the implementation of the program of radical economic transformation in 1989, state control over the food market has been minimized [30].

Our analysis shows significant differences between the national supplies for most food products. Moreover, differences in supply trends were also observed within the countries. Our findings are consistent with many others [31–33] and confirm that governments have a substantial impact on the food available for human consumption and are responsible for ensuring a nutritionally adequate diet. In addition, each country should provide advice on healthy diets in the form of dietary guidelines based on food production, consumption patterns, national public health, nutritional priorities and other factors [5]. Despite the differences between the elements that are taken into account when developing dietary guidelines, both countries recommend differentiated diets and a balance between food intake and physical activity. They are also in line with the World Health Organization’s (WHO’s) three core targets for global reduction: trans- and saturated fats, sodium and sugar [34]. In the Norwegian guidelines the recommended range for total energy from fat is slightly higher than in the Polish recommendations, while total energy recommendations from carbohydrates can be higher for the Polish population. In addition, Polish nutrition recommendations are more precise for saturated fatty acids (SFA), linoleic acid (LA) and alpha-linolenic acid (ALA), but this is consistent also with The Nordic Nutrition Recommendations [6]. Slight differences are also visible in the case of micronutrients and they concern higher recommendation for the intake of vitamin D, B_12_, folate, calcium, magnesium and zinc for the Polish population, with lower intakes of riboflavin, iron, sodium and salt. Greater differences were found when comparing FBDGs. A key message in both countries is to diversify the diet and maintain a balance between food consumption and physical activity, increase the intake of vegetables, fruits, berries, wholegrains and fish, and reduce the intake of fats and processed food. However, they differ when it comes to milk consumption. Poles are recommended to drink two glasses of milk a day, while Norwegians are only recommended to include low-fat milk products in their daily diet. Furthermore, the proportions between the recommended consumption of vegetables and fruit vary. In Poland, it is recommended to eat more vegetables than fruit, while in Norway they should be consumed in comparable amounts. In addition, Norwegian dietary recommendations place emphasis on food labelling – the Keyhole labelling system, which is voluntary for Nordic countries [6]. This helps consumers to choose products that are lower in sugar, fats and salt, and higher in whole grains. There is no such information for consumers in Poland. The Polish recommendations, on the other hand, draw attention to the number of meals and suggest 4–5 meals a day, whereas the Norwegian guidelines do not mention this. Both systems of dietary recommendations have three primary targets: reduction of trans- and saturated fats, sodium and sugar.

Our findings show that the adult diet in both countries matches most of the food products available on the market but does not comply with the FDGs for many of them. Fruit consumption was in line with the recommendation among adult Poles, as the supply of fruit in Poland met the needs of the population in terms of implementing dietary guidelines. However, it should be borne in mind that the link between food availability and food consumption is bi-directional [35]. What is more, the type of food that goes on people’s plate is based on complex preferences and depends on learning experience with food [36]. This is also reflected in our findings. The availability of fish on the Norwegian food market is higher than the country’s recommendations, and only women aged 18–35 were characterized by under-target fish intake. This example indicates that the average availability of food per person does not reflect the actual food intake of different age groups as it does not reflect what people in the country have reported consuming [37]. On the other hand it should be remembered that the balance sheet method serve to assess changes in food availability in a given country, but cannot be directly compared with data obtained from individual consumption surveys, and only allow rough comparisons [38]. Comparisons between the balance sheet method and the consumption surveys should also be treated with caution, as in balance sheet surveys the food availability is calculated per capita of the entire population, while NORKOST 3 and WOBASZ II surveys deal with adult participants. In addition, national food balance does not include food losses due to the wastage at specific points from production to plate [38]. There is therefore a discrepancy between food balance data and individual consumption, which can also be explained by overproduction of certain types of food in relation to demand. This is clearly visible in the case of milk and its products in Poland, where milk production exceeds demand [39, 40]. It is worth mentioning that on the basis of balance sheet research conducted according to Food and Agriculture Organization (FAO) methodology, milk availability in Poland per capita does not differ from the European average [40] and is similar to the data given by the statistical yearbook [15]. For comparison, in Denmark, Ireland and the Netherlands, milk availability is twice as high as in Poland [40]. Therefore, although the supply of milk in Poland is large, this does not translate into a significant increase in consumption, despite the upward trend observed in this study. A different situation with regard to the production and consumption of milk and milk products concerns Norway, where consumption is roughly in line with production. Dairy products are a good source of many macro- and micronutrients, in particular calcium and iodine [41]. As far as this study is concerned, a deficiency of calcium was found in the diet of Poles, which corresponds to lower milk consumption in this country. Thus, despite the availability of dairy products on the food markets of both countries, only in Norway was their consumption satisfactory, which translated into an adequate supply of calcium.

Dietary patterns of both nations differed depending on the consumption of milk and its products, fish and shellfish, which was higher for Norway, and also on the consumption of meat and poultry, vegetables, potatoes, sugar and sweets, and fats, which was higher for Poland. In general, dietary patterns of the Norwegian population tend to be compatible with the Nordic diet recommendations, which emphasize consumption fish and whole grains [42].

According to national statistics and national surveys, the availability and consumption of fish and shellfish in this study is several times lower in Poland, what may depend not only on Poles’ eating habits, but also may be associated with relatively high fish prices on the market as compared to pork and poultry meat [43]. As the statistical trend shows, national supply of these products has been stable for years. Fish and fish liver are an important source of vitamin D. Increasing their consumption in the Polish population may reduce to some extent the deficit of this vitamin [44]. However, as the example of Norway in our study shows, despite significantly higher fish consumption and higher levels of vitamin D in the diet compared to Poland, the deficiency problem still exists. In Norway, the health authorities recommend a daily vitamin D supplement from the age of 4 weeks [45]. In Poland, supplementation of vitamin D is recommended from the first days of life. Polish recommendations also point to diet as a source of vitamin D, and in particular to fish such as eel, wild salmon and herring [46].

Both national statistics and surveys showed that sugar and sweet product availability and consumption was higher in Poland compared to Norway. In addition, an unfavorable tendency to increase consumption of these products has been noted in Poland, and in Norway this trend has decreased. The situation is critical, because three out of five adult Poles are overweight and one in four is obese [47]. In Poland the tendency to sweeten drinks and dishes is decreasing, but at the same time there is more sugar added to processed food [48]. Therefore, its total consumption is increasing. The intake of free sugars, which in the case of Norway did not exceed 10% of energy, is in line with WHO recommendations [49]. In contrast, in the Polish population, sugar consumption was significantly higher than in Norway and exceeded 10%. Currently, the Polish authorities are increasing the tax on sugar, as previously done in Norway.

This study has some strengths and limitations. The strong point of this study is its representativeness for the Norwegian and Polish populations, a similar methodology for evaluating nutrition and a large coverage of food products taken into account. As regards limitations, firstly, food consumption depends not only on the amount of food on the market, whether the quantity and variety of food is regulated by individual countries, but also on the eating habits of citizens, which may influence the study outcomes. In addition, the weights of individual products were taken into account for the calculation of milk and milk products intake without conversion into the quantity of milk intended for the production of these products, which could have affected the final data. Mild to moderate iodine deficiency has re-emerged as a public health concern in Norway [50]. However, NORKOST3 has no data on iodine, thus iodine intake has not been addressed.

## Conclusions

This study concluded that state intervention in the food market does not restrict its access for citizens. Despite some diversity restrictions, the diet of Norwegian citizens was better balanced in terms of food and micronutrient content. The authorities of both countries should draw attention to the fact that a good availability of various groups of food does not reduce the problem of widespread dietary deficiency in vitamin D and folic acid, and action should be taken at national level to eliminate their inadequacy. In the light of increasing risk of non-communicable diseases, low compliance with the dietary guidelines for milk, fish and sugar consumption in Poland requires specific educational campaigns aimed at increasing dietary literacy in vulnerable groups.

## Supplementary Information

**Additional file 1: Table S1.** Polish and Norwegian recommendations of macronutrients intake for adults (18–64 years). **Table S2.** Polish and Norwegian recommendation of micronutrients for adults (18–64 years). **Table S3.** Polish and Norwegian recommendation of vitamins for adults (18–64 years). **Table S4.** Comparison of national dietary recommendations for adults in Norway and Poland.

## Data Availability

The datasets used and analysed during the current study are available from the corresponding author on reasonable request. WOBASZ II data was obtained on permission from Department of Epidemiology, Cardiovascular Disease Prevention and Health Promotion, National Institute of Cardiology, Warsaw, Poland. NORKOST 3 data was obtained by permission from University of Oslo.

## Abbreviations

AI: Adequate Intake
ALA: Alpha-linolenic acid
E%: % of energy intake
EFSA: European Food Safety Authority
EEA: European Economic Area
EU: European Union
FAO: Food and Agriculture Organization
FBDGs: Food based dietary guidelines
LA: Linoleic acid
MUFA: Monounsaturated fatty acids
PUFA: Polyunsaturated fatty acids
RDA: Recommended Dietary Allowance
RI: Recommended intake
SD: Standard deviation
SFA: Saturated fatty acids
WHO: World Health Organization

## References

[1] Global Nutrition Report 2017. http://globalnutritionreport.org/the-report. Accessed 8 May 2018.

[2] Nordic food systems for improved health and sustainability. Baseline assessment to inform transformation. Stockholm Resilience Centre. March 2019. http://pure.iiasa.ac.at/id/eprint/16122/1/SRC_Report%20Nordic%20Food%20System.pdf. Accessed 12 March 2020.

[3] White RO, Thompson JR, Rothman RL, McDougald Scott AM, Heerman WJ, Sommer EC (2013). A health literate approach to the prevention of childhood overweight and obesity. Patient Educ Couns.

[4] Health Promotion & Disease Prevention. Nutrition. https://ec.europa.eu/jrc/en/health-knowledge-gateway/promotion-prevention/nutrition. Accessed 3 April 2019.

[5] Health Promotion & Disease Prevention. Food-Based Dietary Guidelines in Europe. https://ec.europa.eu/jrc/en/health-knowledge-gateway/promotion-prevention/nutrition/food-based-dietary-guidelines. Accessed 5 April 2019.

[6] The Nordic Nutrition Recommendation 2012 (2014). Integrating nutrition and physical activity.

[7] World Health Organization (2014). Action - Norwegian guidelines on diet, nutrition and physical activity. Dietary goals and food-based dietary guidelines - all population groups.

[8] Jarosz M (2017). Nutrition standards for polish population [in polish].

[9] Morland K, Wing S, Diez RA (2002). The contextual effect of the local food environment on residents’ diets: the atherosclerosis risk in communities study. Am J Public Health.

[10] Roos G, Leon M, Anderson A (2002). Dietary interventions in Finland, Norway and Sweden: nutrition policies and strategies. J Hum Nutr Diet.

[11] Ralston K, Frazao E (1999). How government policies and regulations can affect dietary choice. Americans eating habits: changes and consequences, agriculture information bulletin no. (AIB-750) 494 pp. U.S. Department of Agriculture USDA/ERS.

[12] U.S. Department of Agriculture and U.S. Department of Health and Human Services (2010). Dietary guidelines for Americans.

[13] EEA Agreement. Protocol 3 concerning products referred to in article 8(3) (b) of the agreement. https://www.efta.int/media/documents/legal-texts/eea/the-eea-agreement/Protocols%20to%20the%20Agreement/protocol3.pdf. Accessed 29 September 2020.

[14] European Economic Area Joint Committee. 4 November 2016 3 Annexes Ref. 16-4665 Annual Report of the EEA Joint Committee 2015. The Functioning of the EEA Agreement (Article 94(4)). https://www.efta.int/sites/default/files/documents/eea/eea-institutions/Joint%20Committee%20Annual%20Report%202015.pdf. Accessed 29 September 2020.

[15] Statistics Poland. Statistical yearbooks. https://stat.gov.pl/en/topics/statistical-yearbooks. Accessed 23 May 2018.

[16] The Norwegian Directorate for Health. Untviklingen i norsk kosthold. https://helsedirektoratet.no/publikasjoner/utviklingen-i-norsk-kosthold. Accessed 28 May 2018.

[17] FAO. Technical conversion factors for agricultural commodities. http://www.fao.org/economic/the-statistics-division-ess/methodology/methodology-systems/technical-conversion-factors-for-agricultural-commodities/ar. Accessed 7 April 2019.

[18] Szczygieł A, Nowicka L, Bułhak-Jachymczyk B (1987). Normy żywienia i wyżywienia. Cz. II. Normy wyżywienia – modele racji pokarmowych [in polish].

[19] Totland TH, Melnæs BK, Lundberg Hallén N, Helland-Kigen KM, Lund-Blix NA, Myhre JB, et al. Norkost 3. En landsomfattende kostholdsundersøkelse blant menn og kvinner i Norge i alderen 18–70 år, 2010–2011. 2012. https://www.helsedirektoratet.no/rapporter/norkost-3-en-landsomfattende-kostholdsundersokelse-blant-menn-og-kvinner-i-norge-i-alderen-18-70-ar-2010-11. Accessed 7 April 2019.

[20] Myhre JB, Loken EB, Wandel M, Andersen LF (2013). Eating location is associated with the nutritional quality of the diet in Norwegian adults. Public Health Nutr.

[21] Waśkiewicz A, Szcześniewska D, Szostak-Węgierek D, Kwaśniewska M, Pająk A, Stepaniak U (2016). Are dietary habits of the polish population consistent with the recommendations for prevention of cardiovascular disease? - WOBASZ II project. Kardiol Pol.

[22] Drygas W, Niklas AA, Piwońska A, Piotrowski W, Flotyńska A, Kwaśniewska M (2016). Multi-center National Population Health Examination Survey (WOBASZ II study): assumptions, methods and implementation. Kardiol Pol.

[23] Kunachowicz H, Nadolna I, Przygoda B, Iwanow K (2005). Tables of nutritional value of food products and dishes3rd edition extended and updated.

[24] Norwegian Food Composition Table (FCT). https://www.norge.no/en/service/norwegian-food-composition-table-fct. Accessed 18 June 2019.

[25] European External Action Service website. EU and Norway Trade Relations. http://eeas.europa.eu/delegations/norway/eu_norway/trade_relation/index_en.htm Assessed 23 June 2019.

[26] OECD. Agricultural support (indicator). 10.1787/6ea85c58-en. Accessed 6 March 2020.

[27] Hemmings P. Policy challenges for agriculture and rural areas in Norway. OECD Economics Department Working Papers, No. 1286, OECD Publishing, Paris, 2016. 10.1787/5jm0xf0r676c-en. Assessed 6 March 2020.

[28] Brooks J, Matthews A. Trade dimensions of food security. Paris: OECD Food, Agriculture and Fisheries Papers, No. 77, OECD Publishing. 10.1787/5js65xn790nv-en. Assessed 6 March 2020

[29] Report from the Nordic competition authorities. Nordic Food Markets – a taste for competition. http://www.konkurrensverket.se/globalassets/english/publications-and-decisions/nordic-food-markets%2D%2Da-taste-for-competition.pdf. Assessed 20 February 2020.

[30] Henson S, Sekuła W (1994). Market reform in the polish food sector: impact upon food consumption and nutrition. Food Policy.

[31] Kearney J (2010). Food consumption trends and drivers. Philos Trans R Soc Lond Ser B Biol Sci.

[32] Thow AM (2009). Trade liberalization and nutrition transition: mapping the pathways for public health nutritionists. Public Health Nutr.

[33] Fallows SJ, Wheelock JV (1985). Role of government in food supply. Nutr Health.

[34] GBD 2017 Diet Collaborators (2019). Health effects of dietary risks in 195 countries, 1990–2017: a systematic analysis for the global burden of disease study 2017. Lancet.

[35] Herford A, Ahmed S (2015). The food environment, its effects on dietary consumption, and potential for measurement within agriculture-nutrition interventions. Food Secur.

[36] Capaldi ED, Capaldi ED (1996). Conditioned food preferences. Why we eat what we eat: the psychology of eating.

[37] Marriott BP, Fink CJ, Krakower T, Rippe JM (2014). Worldwide consumption of sweeteners and recent trends. Fructose, high fructose corn syrup, sucrose and health. Nutrition and health.

[38] Serra-Majem L, MacLean D, Ribas L, Brulé D, Sekula W, Prattala R (2003). Comparative analysis of nutrition data from national, household, and individual levels: results from a WHO-CINDI collaborative project in Canada, Finland, Poland, and Spain. J Epidemiol Community Health.

[39] Seremak-Bulge J, Bodył M (2014). Milk consumption in Poland compared to other countries. Probl Agric Econom.

[40] Bórawski P, Dunn JW (2015). Differentiation of milk production in European Union countries in the aspect of common agricultural policy. Roczniki Naukowe Stowarzyszenia Ekonomistów Rolnictwa i Agrobiznesu.

[41] Nerhus I, Wik Markhus M, Nilsen BM, Øyen J, Maage A, Ødegård ER, et al. Iodine content of six fish species, Norwegian dairy products and hen’s egg. Food Nutr Res. 2018;62. 10.29219/fnr.v62.1291 PMC5971469 29853825

[42] Adamsson V, Reumark A, Fredriksson IB, Hammarström E, Vessby B, Johansson G (2011). Effects of a healthy Nordic diet on cardiovascular risk factors in hypercholesterolaemic subjects: a randomized controlled trial (NORDIET). J Intern Med.

[43] Ruciński P. Fish and Seafood Market in Poland. USDA Foreign Agricultural Service. USDA Foreign Agricultural Service. Global Agricultural Information Network. https://apps.fas.usda.gov/newgainapi/api/report/downloadreportbyfilename?filename=2017%20Fish%20and%20Seafood%20Market%20in%20Poland_Warsaw_Poland_2-21-2018.pdf. Accessed 24 February 2020.

[44] Malesa-Ciećwierz M, Usydus Z (2015). Vitamin D: can fish food-based solutions be used for reduction of vitamin D deficiency in Poland?. Nutrition.

[45] D-vitamin: Anbefalt inntak er 10 mikrogram (μg) per dag for barn i alderen 6–11 måneder. Fra spedbarn er fire uker gamle, anbefales D-vitamintilskud. https://www.helsedirektoratet.no/retningslinjer/spedbarnsernaering/anbefalinger-for-tilforsel-av-energi-og-naeringsstoffer-til-spedbarn-611-maneder/d-vitamin-anbefalt-inntak-er-10-mikrogram-g-per-dag-for-barn-i-alderen-611-maneder-fra-spedbarn-er-fire-uker-gamle-anbefales-d-vitamintilskudd. Assessed 12 March 2020.

[46] Rusinska A, Pludowski P, Walczak M, Borszewska-Kornacka M, Bossowski A, Chlebna-Sokół D (2018). Vitamin D supplementation guidelines for general population and groups at risk of vitamin D deficiency in Poland—recommendations of the polish Society of Pediatric Endocrinology and Diabetes and the expert panel with participation of national specialist consultants and representatives of scientific societies—2018 update. Front Endocrinol (Lausanne).

[47] Stepaniak U, Micek A, Waśkiewicz A, Bielecki W, Drygas W, Janion M (2016). Prevalence of general and abdominal obesity and overweight among adults in Poland. Results of the WOBASZ II study (2013-2014) and comparison with the WOBASZ study (2003-2005). Pol Arch Med Wewn.

[48] National Health Fund. [In Polish: Raport „Cukier, otyłość – konsekwencje. Przegląd literatury, szacunki dla Polski”] Report “Sugar, obesity – consequences. Literature review, estimates for Poland”. https://www.nfz.gov.pl/download/gfx/nfz/pl/defaultstronaopisowa/349/43/1/raport_-_cukier.pdf . Accessed 23 September 2020.

[49] World Health Organization. Guideline: sugars intake for adults and children. https://www.who.int/nutrition/publications/guidelines/sugars_intake/en. Assessed 5 February 2020. 25905159

[50] Brantsæter AL, Abel MH, Haugen M, Meltzer HM (2013). Risk of suboptimal iodine intake in pregnant Norwegian women. Nutrients.

