# Supplementary material for: Diet quality in the population of Norway and Poland: differences in the availability and consumption of food considering national nutrition guidelines and food market
Source: BMC Public Health. 2021 Feb 9;21:319. doi: 10.1186/s12889-021-10361-3 (PMC7871600; doi:10.1186/s12889-021-10361-3)
Supplement: Supplementary file 1 — Additional file 1: Table S1. Polish and Norwegian recommendations of macronutrients intake for adults (18–64 years). Table S2. Polish and Norwegian recommendation of micronutrients for adults (18–64 years). Table S3. Polish and Norwegian recommendation of vitamins for adults (18–64 years). Table S4. Comparison of national dietary recommendations for adults in Norway and Poland. [file 12889_2021_10361_MOESM1_ESM.docx]

**Additional files**

**Table 1** Polish and Norwegian recommendations of macronutrients intake for adults (18-64 years)

| Nutrition recommendations | | | Poland^1^ | Norway^2^ |
| --- | --- | --- | --- | --- |
| MACRONUTRIENTS | Fatty acids | Total | 20-35E% | 25-40E% |
|  |  | SFA | 5-6 E% | < 10 E% |
|  |  |  |  |  |
|  |  | TFA | as low as possible** | as low as possible |
|  |  | LA+ALA | 4E% | > 3E% |
|  |  | ALA | 0.5E% | 0.5E% |
|  | Protein |  | 10-20E% | 10-20E% |
|  | Carbohydrates | Total | 45-65E% | 45-60E% |
|  |  | Dietary fibre | 25g/d * | > 25-35 g/d |
|  |  | Added sugar | < 10 E% | < 10 E% |

Data are presented as recommended intake (RI) for adults (18-64 years) both gender

^*^AI Adequate Intake SFA- saturated fatty acids, TFA- trans fatty acids, LA- linoleic acid, ALA- alpha-linolenic acid

**The value should be as low as possible in a diet that provides the right nutritional value

^1^ Nutrition standards for Polish population 2017

^2^ Nordic Nutrition recommendations 2012

**Table 2** Polish and Norwegian recommendation of micronutrients for adults (18-64 years)

| Nutrition recommendations | | Poland^1^ | Norway^2^ |
| --- | --- | --- | --- |
| MICRONUTRIENTS | Iron | F 6-8 mg/d*  M 6 mg/d | F 9-15mg/d  M 9-11 mg/d |
|  | Magnesium | F 310-320 mg/d*  M 400-420 mg/d* | F 280 mg/d  M 350 mg/d |
|  | Potassium** | 3.5 g/d | F 3.1 g/d  M 3.5 g/d |
|  | Calcium | F 1000-1200 mg/d*  M 1000 mg/d | 800 mg/d |
|  | Zinc | F 8 mg/d  M 11 mg/d | F 7 mg/d  M 9 mg/d |
|  | Selenium | 55 µg/d | F 50 µg/d  M 60 µg/d |
|  | Iodine | 150 µg/d | 150 µg/d |
|  | Sodium** | 1.4-1.5 g/d * | 2.4 g/d |
|  | Salt*** | 5 g/d | 6 g/d |

Data are presented as recommended intake (RI) for adults (18-64 years) both gender

^1^ Nutrition standards for Polish population 2017

^2^ Nordic Nutrition recommendations 2012

* depending on age group

** expressed as AI (Adequate Intake)

*** expressed as maximum recommended intake.

**Table 3** Polish and Norwegian recommendation of vitamins for adults (18-64 years)

| Nutrition recommendations | | Poland^1^ | Norway^2^ |
| --- | --- | --- | --- |
| VITAMINS | D* | 15µg/d | 10µg/d |
|  | B6 | F 1.3-1.5mg/d**  M 1.3-1.7mg/d** | F 1.2 -1.3mg/d  M 1.5mg/d |
|  | B12 | 2.4µg/d | 2µg/d |
|  | Thiamine | F 1.1mg/d  M 1.3mg/d | F 1.0-1.1mg/d  M 1.2-1.4mg/d |
|  | Riboflavin | F 1.1mg/d  M 1.3mg/d | F 1.2-1.3mg/d  M 1.4-1.6mg/d |
|  | Folate | F 450µg/d  M 400 µg/d | F 300-400µg/d  M 300µg/d |
|  | A, RAE | F 700µg/d  M 900µg/d | F 700µg/d  M 900µg/d |
|  | E,  α-Tocopherol* | F 8mg/d  M 10mg/d | F 8mg/d  M 10mg/d |
|  | C | F 75mg/d  M 90mg/d | 75mg/d |

Data are presented as recommended intakes (RI) for adults (18-64 years) both gender

^1^ Nutrition standards for Polish population 2017

^2^ Nordic Nutrition recommendation

* expressed as AI (Adequate Intake)

** depending on age group.

**Table 4** Comparison of national dietary recommendations for adults in Norway and Poland*

| Food group | Norway^1^ | Poland^2^ |
| --- | --- | --- |
|  |  |  |
| Milk and milk products | Include low fat dairy products in your daily diet. Limit the use of dairy products with a lot of saturated fats like whole milk, cream, fat cheese and butter and those with high amount of salt and added sugar. | Drink at least 2 large glasses of milk a day. Milk can be replaced by yoghurt, kefir and some cheese. |
| Poultry and meat | Choose lean meat products. Limit the amount of processed meat and red meat to 0.5 kg per week. Choose white and low fat meat products. | Eat meat in moderation (reduce red and processed meat products consumption to 0.5 kg per week).  1-2 times a week it is worth changing meat for a dish of soybeans, peas, beans, lentils or eggs. |
| Fish and shellfish | Eat fish 2-3 times a week (300-450 grams of pure fish per week, at least 200 grams should be fatty fish as salmon, trout, mackerel or herring). Use also fish as a spread on bread. | Eat fish at least twice a week (mainly saltwater fish). However, it must not be fried fish, but baked and cooked. |
| Cereals inc. rice | Eat whole grain foods every day. Choose grain products with high fiber and whole grain and low fat and sugar. The whole grain products should be consumed in the amount of 70-90 grams of combined flour or whole grain per day. | Cereals products should be your principal source of calories, consume particularly whole grain products. |
| Vegetables, friuts and berries | Consume at least 500 grams of vegetables, fruits and berries a day in 5 portions. The proportion should be ½ vegetables and ½ fruits and berries.  You can use fresh, hermetically, frozen and heat-treated vegetables, fruits and berries. Choose between different types of vegetables and fruits.  Eat the seeds of legumes such as beans, lentils as well as spices and herbs although they are not included in five a day but belong to varied diet. | Vegetables and fruits should represent at least half of what you eat. Remember about the right proportions of ¾ vegetables and ¼ fruits. Consume at least 400 grams a day in 5 portions (one portion can be a glass of juice). Eat the seeds of legumes (mainly beans, lentils, green peas and broad beans). |
| Potatoes | Belong to a varied diet. You can choose between boiled or baked potatoes | - |
| Nuts | Eat a small handful of unsalted nuts a day | Eat nuts instead of sweets |
| Sugar and sweet products | Avoid foods and drinks that are high in sugar | Limit your intake of sugar and sweets, replace them with fruits and nuts. |
| Margarine, butter, oil etc | Choose edible oils, liquid margarine and soft margarine spreads instead of hard margarines and butter. | Limit the consumption of animal fats, replace them with vegetable oils. |
| Salt | Choose food that are low in salt and limit the use of salt when preparing food and at the table. | Limit salt intake. Do not treat dishes with salt, buy products with a low salt content. Use herbs, they have valuable ingredients and improve the taste. |
|  |  |  |
| Water | Choose water as a thirst-quencher. | Drink at least 1,5 liter of water a day |
| Meal regularity | - | Eat regular meals (4-5 meals every 3-4 hour) Breakfast should be eaten 1-2 hours after getting up, supper 3 hours before bedding. |
| Physical activity | Be physically active for at least 30 minutes each day | Be physical active at least 30-45 minutes each day |
| Nutrition labeling | Look for the Keyhole when shopping for food | - |
| Key nutrients targeted | Trans and saturated fats, salt and sugar | Trans and saturated fats, salt and sugar |

^1^ Recommendation about diet, nutrition and physical activity 2014

^2^ Nutrition standards for Polish population 2017

*Recommendations are addressed to healthy people to maintain good health.
